# Beneficial microorganisms: Regulating growth and defense for plant welfare

**DOI:** 10.1111/pbi.14554

**Published:** 2024-12-20

**Authors:** Yan Liu, Aiqin Shi, Yue Chen, Zhihui Xu, Yongxin Liu, Yanlai Yao, Yiming Wang, Baolei Jia

**Affiliations:** ^1^ Xianghu Laboratory Hangzhou China; ^2^ Jiangsu Provincial Key Lab of Solid Organic Waste Utilization Nanjing Agricultural University Nanjing China; ^3^ Horticulture Research Institute Zhejiang Academy of Agricultural Sciences Hangzhou China; ^4^ Shenzhen Branch, Guangdong Laboratory of Lingnan Modern Agriculture, Genome Analysis Laboratory of the Ministry of Agriculture and Rural Affairs, Agricultural Genomics Institute at Shenzhen Chinese Academy of Agricultural Sciences Shenzhen China; ^5^ Institute of Environment, Resource, Soil and Fertiliser Zhejiang Academy of Agricultural Sciences Hangzhou China; ^6^ Department of Plant Pathology, Key Laboratory of Integrated Management of Crop Diseases and Pests, Ministry of Education Nanjing Agricultural University Nanjing China

**Keywords:** Growth‐defense trade‐offs, beneficial microorganisms, rhizosphere microbiomes, biotic stresses, abiotic stresses

## Abstract

Beneficial microorganisms (BMs) promote plant growth and enhance stress resistance. This review summarizes how BMs induce growth promotion by improving nutrient uptake, producing growth‐promoting hormones and stimulating root development. How BMs enhance disease resistance and help protect plants from abiotic stresses has also been explored. Growth‐defense trade‐offs are known to affect the ability of plants to survive under unfavourable conditions. This review discusses studies demonstrating that BMs regulate growth‐defense trade‐offs through microbe‐associated molecular patterns and multiple pathways, including the leucine‐rich repeat receptor‐like kinase pathway, abscisic acid signalling pathway and specific transcriptional factor regulation. This multifaceted relationship underscores the significance of BMs in sustainable agriculture. Finally, the need for integration of artificial intelligence to revolutionize biofertilizer research has been highlighted. This review also elucidates the cutting‐edge advancements and potential of plant‐microbe synergistic microbial agents.

## Introduction

Plants undergo biotic and abiotic stresses in their life cycles. Since plants harbour a limited pool of nutrient resources, alteration in their nutrient utilization is essential for adaptation and survival under unfavourable conditions such as stress (Deng *et al*., 2020; Gao *et al*., 2021). To optimize resource allocation, plants have evolved strategies for balancing their limited resources between growth and defense, referred to as growth‐defense trade‐offs (He *et al*., 2022). Specific intercellular factors influence these balancing processes, including phytohormones, the mitogen‐activated protein kinase (MAPK) pathway, post‐transcriptional modifications and the circadian oscillator (He *et al*., 2022; Liu, Xu *et al*., 2022; Shields *et al*., 2022). Among the phytohormones, auxin (AUX), brassinosteroid (BR), cytokinin (CTK) and gibberellin (GA) affect the growth module, whereas salicylic acid (SA), jasmonic acid (JA) and ethylene (ET) influence the defense module (He *et al*., 2022). The hormones and their receptors, such as GID1 for GA (Hirano *et al*., 2008), COI1 for JA (Liu *et al*., 2021), TIR1 for AUX (Lu *et al*., 2024), PYR/PYL/RCAR for abscisic acid (ABA) (Gonzalez‐Guzman *et al*., 2012) and ETR1 for ET (Zdarska *et al*., 2019), affect the growth‐defense balance. Pattern recognition receptors (PRRs) directly recognize specific molecular structures on the surfaces of pathogens and participate in growth‐defense trade‐offs (Li and Wu, 2021).

Rhizosphere microbiomes have co‐evolved with their hosts to optimize nutrient acquisition and immune responses (Bakker *et al*., 2018). Plant roots can secrete abundant exudates, including amino acids, organic acids, soluble sugars, fatty acids and sterols. And these exudates dynamically alter the composition of the rhizosphere microbiota and recruit beneficial microorganisms (in the following, we use the abbreviation BMs instead) (Chepsergon and Moleleki, 2023; Mommer *et al*., 2016; Vives‐Peris *et al*., 2020; Zhao *et al*., 2024). BMs further promote plant growth and enhance plant resistance to pathogens (Figueroa‐Macías *et al*., 2021; Löser *et al*., 2021; Metcalf and Koskella, 2019; Monson *et al*., 2022). Several BMs have been commercialized, with various species and strains serving as biofertilizers and biocontrol agents. These microorganisms exhibit diverse functions and mechanisms to promote crop growth and protect against biotic and abiotic stresses.

Under stress, BMs stimulate plant recruitment signals and regulate energy allocation to growth and defense via different mechanisms. Therefore, understanding the role of the plant rhizosphere in the growth‐defense trade‐offs of plants can provide valuable insights into how microbial interactions can be manipulated to enhance crop resilience and productivity. The mechanisms underlying plant growth‐defense trade‐offs can help illustrate how plants respond to environmental challenges and manage resources to ensure survival and reproduction and have been extensively summarized in previous reviews (Dardanelli *et al*., 2008; He *et al*., 2022; Huot *et al*., 2014; Monson *et al*., 2022). To elucidate the functions of BMs in plant growth, defense, and their trade‐offs, this review discusses the current research on the following aspects: the role of BMs in promoting plant growth; regulatory patterns of BMs against biotic and abiotic stresses; and mechanisms by which BMs regulate growth‐defense trade‐offs in plants. The review concludes with future perspectives for research and agricultural applications.

## BMs induce growth promotion in plants

**Figure 1:**
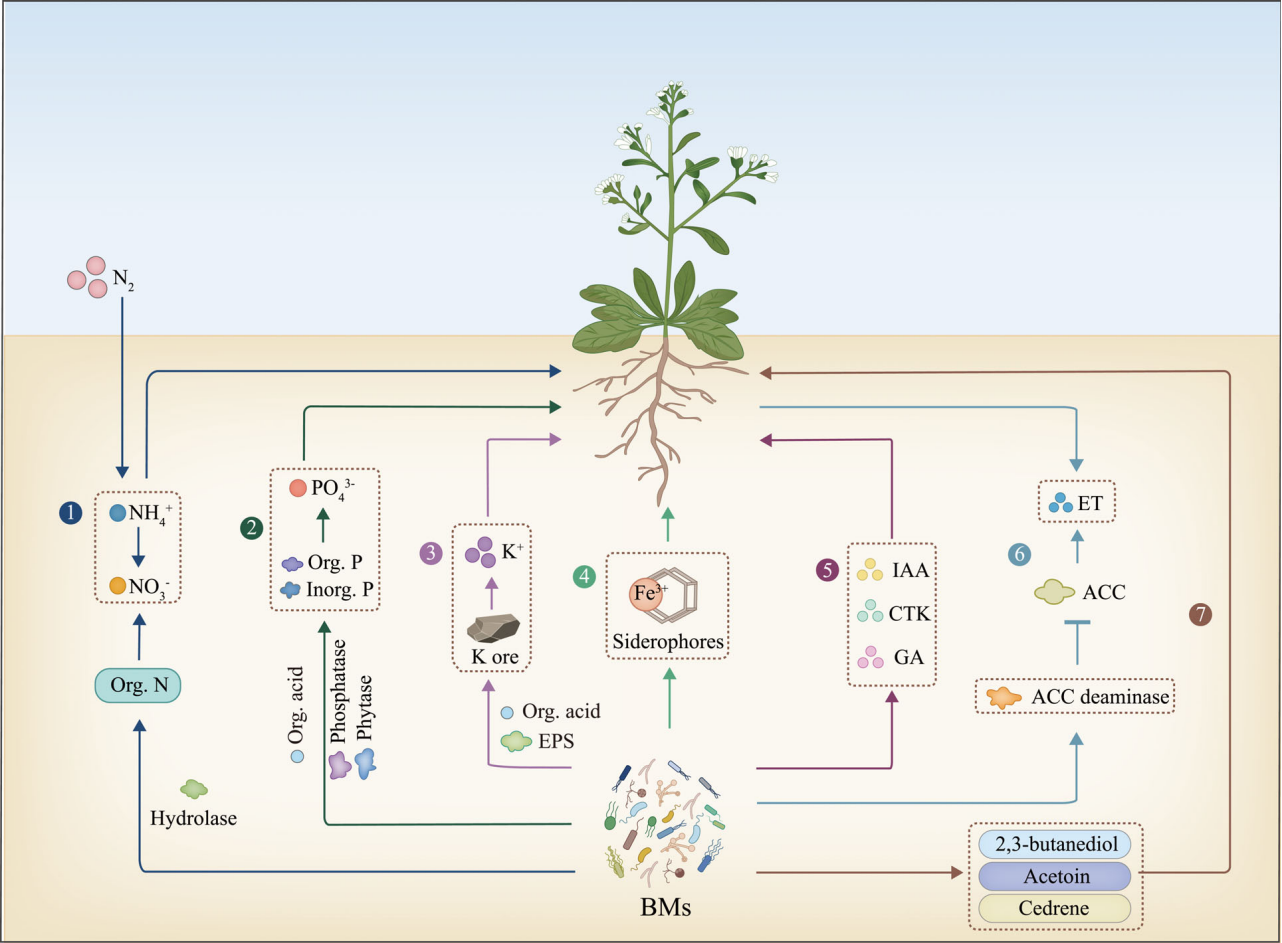
Mechanisms by which beneficial microbiomes (BMs) prompt plant growth and development. BMs convert nitrogen from the air into ammonium and secrete hydrolases to degrade organic nitrogen into nitrate (①); BMs secrete organic acids, phytase, and phosphatase to dissolve insoluble phosphorus (②); BMs secrete organic acids and exopolysaccharides (EPS) to decompose insoluble potassium (③); BMs secrete siderophores to chelate iron ions around the rhizosphere (④); BMs produce various plant growth‐related hormones to promote plant growth (⑤); BMs produce aminocyclopropane‐1‐carboxylate (ACC) deaminase to degrade ACC, the precursor of ET (⑥); BMs produce VOCs (such as 2,3‐butanediol, acetoin, and cedrene) to modulate plant growth (⑦). ET, Ethylene; ACC, 1‐aminocyclopropane‐1‐carboxylate; VOCs, Volatile organic compounds.

BMs play a key role in promoting plant growth. BMs can improve rhizosphere nutrient availability, increase nutrient uptake and nitrogen fixation, and induce the secretion of hormones that promote plant growth and development (Figure 1). Many essential nutrients that cannot be directly used by plants, including nitrogen (N), phosphorus (P) and potassium (K), can be transformed into usable forms through BMs; this transformation helps root absorption and utilization. For example, *Azospirillum* spp. converts N_2_ into ammonium which fertilizes plants. Nitrifying bacteria metabolize ammonium into nitrite and nitrate to improve N availability to plants (Dardanelli *et al*., 2008; Huot *et al*., 2014). Arbuscular mycorrhizal fungi (AMF) secrete organic acids to dissolve insoluble P (Zhou *et al*., 2022). *Bacillus* spp. decompose organic and inorganic P by secreting phytase and phosphatase (Saeid *et al*., 2018). *B. velezensis* and *B. coagulans* secrete organic acids and exopolysaccharides (EPSs) to dissolve insoluble K, thus improving K availability to plants (Meena *et al*., 2014). The secretion of iron chelator siderophores from BMs also improves iron utilization in plants (Subramanium and Sundaram, 2020). BMs belonging to *Pseudomonas*, *Klebsiella*, *Bacillus*, *Bradyrhizobium*, *Streptomyces*, *Serratia* and *Rhizobium* secrete siderophores (Mustafa *et al*., 2019), suggesting that improving iron homeostasis is a common mechanism by which BMs improve plant growth.

Phytohormones are required for different physiological processes in plants. Plant‐associated BMs produce various plant growth‐related hormones, such as indole acetic acid (IAA), CTK and GA, to directly promote plant growth. Rhizosphere‐colonized *Pseudomonas* and *Rhizobium* produce IAA, which can promote main root elongation and increase root surface area, enhancing nutrient and water absorption (Luo *et al*., 2018; Ma *et al*., 2022). In *Arabidopsis*, UBC13 is affecting auxin signalling and Aux/IAA protein stability. The primary root of ubc13 double mutant was about one‐third of the WT in length on day 5 after seed planting (Wen *et al*., 2014). *Bacillus*, *Escherichia*, *Agrobacterium* and *Methylobacterium* secrete CTK, which significantly influences plant seed germination, root elongation and vascular development (Maheshwari *et al*., 2015). GAs, secondary metabolites produced by *Leifsonia soli*, positively affect growth in cucumbers, radishes and tomatoes (Kang *et al*., 2014). Similarly, rhizosphere microorganisms, such as *B. circulans* and *B. firmus*, produce 1‐aminocyclopropane‐1‐carboxylate (ACC) deaminase and promote ACC efflux from plant roots (Gamalero and Glick, 2015; Ghosh *et al*., 2003), decreasing ET production to enhance growth (Nadeem *et al*., 2010). Volatile organic compounds (VOCs) secreted by *B. subtilis*, *B. amyloliquefaciens* and *B. velezensis* also promote plant root development (Li *et al*., 2021, 2022; Ryu *et al*., 2004).

## BMs protect against biotic and abiotic stresses in plants

Besides promoting plant growth, BMs play a crucial role in fortifying plants against pathogens and diseases (biotic stresses) by producing antimicrobial compounds and activating immune responses. BMs also help plants withstand drought, salinity and other abiotic stresses by improving water and nutrient uptake, modulating stress‐related pathways and promoting root development. The following section specifically elucidates the roles of BMs in mitigating biotic and abiotic stresses.

### BMs enhance disease resistance in plants

**Figure 2:**
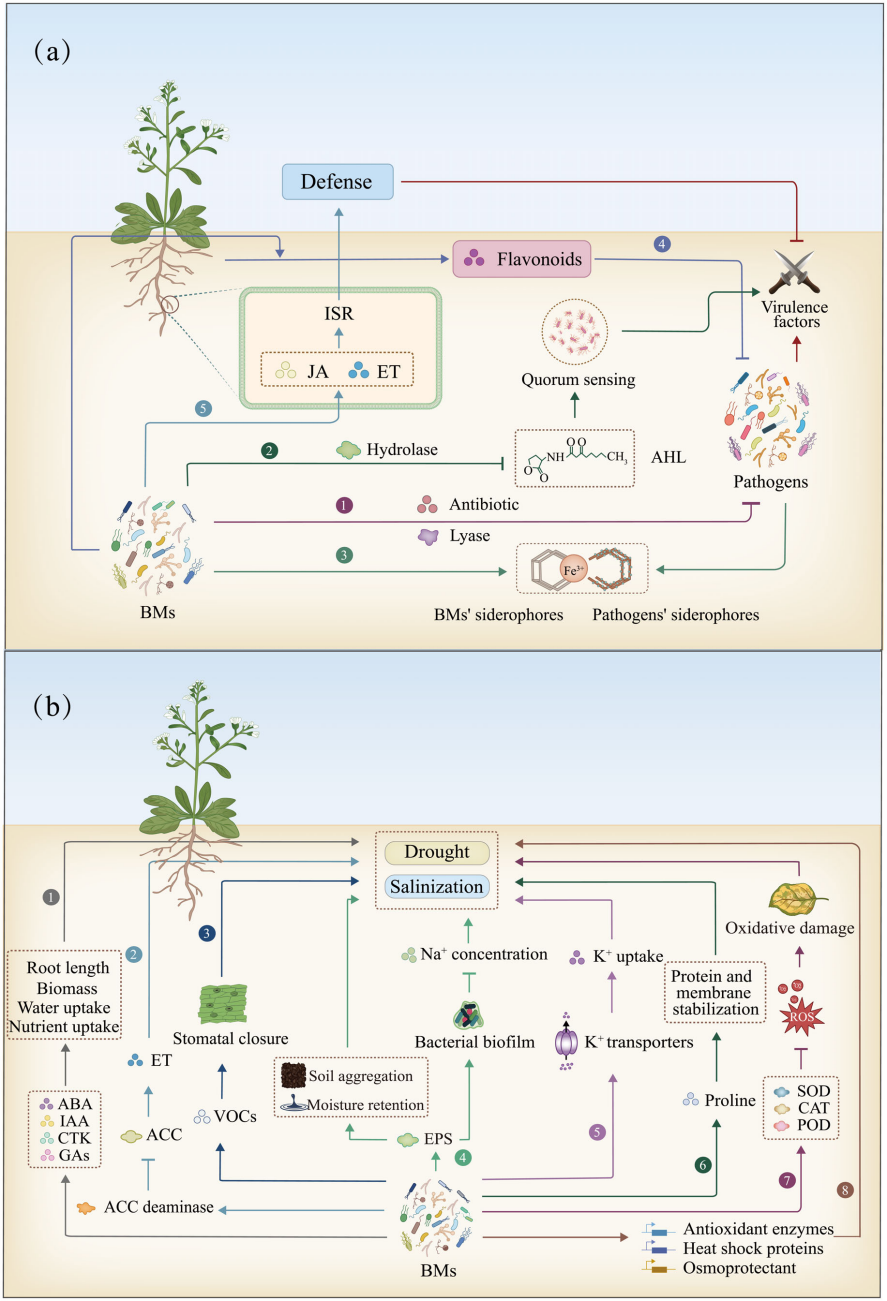
BMs help plants counter biotic and abiotic stresses via multiple mechanisms. (a) BMs antagonize pathogens through multiple pathways: BMs produce antibiotics and lyases which inhibit and/or kill pathogenic microorganisms (①); BMs secrete hydrolase to degrade AHL, blocking quorum sensing between pathogens (②); BMs secrete high‐affinity siderophores to sequester iron ions which are normally chelated by low‐affinity siderophores secreted by pathogens (③); BMs induce plants to biosynthesize bacteriostatic compounds (④); BMs recognize microbe‐associated molecular patterns (MAMPS) to activate ISR in plants (⑤). (b) BMs counter abiotic stresses (e.g. drought and salinization) through multiple mechanisms: BMs produce various compounds to promote plant growth and enhance resistance (①); BMs produce ACC deaminase to reduce ET levels in plants (②); BMs produce VOCs to induce air holes, reducing water loss (③); BMs produce EPS, which improves soil aggregation and moisture. EPS promotes biofilm formation to capture and prevent Na^+^ from entering the plant (④); BMs adjust Na^+^ into liquid bubbles to help plants maintain ion homeostasis (⑤); BMs regulate the expression of ion‐transferring protein, enhancing the absorption of K^+^ (⑥); BMs induce substance accumulation in plants to stabilize proteins and membranes (⑦); BMs stimulate plants to produce antioxidant enzymes, remove reactive oxygen species (ROS), and reduce plant oxidative damage (⑧); BMs increase the expression of genes related to stress tolerance and enhance plant drought resistance (⑨). ISR, Induced systemic resistance; JA, Jasmonic acid; ET, Ethylene; AHL, N‐acyl‐homoserine lactone. ET, Ethylene; ACC, 1‐aminocyclopropane‐1‐carboxylate; VOCs, Volatile organic compounds; EPS, Exopolysaccharide; ROS, reactive oxygen species; SOD, Superoxide dismutase; CAT, Catalase; POD, Peroxidase.

Pathogenic infections are the major biotic stress in plants (Suzuki *et al*., 2014). Various mechanisms have been developed by BMs to antagonize pathogens, including secreting secondary metabolites to kill pathogens, interrupting quorum sensing (QS) among pathogens, producing siderophores to compete for iron, inducing plants to produce antibacterial substances and activating resistance systems (Figure 2a).

Direct killing is a major mechanism by which plants resist pathogens. *Pseudomonas* secretes 2,4‐diacetylphloroglucinol, a broad‐spectrum antibiotic that can protect tomato plants against *Fusarium oxysporum* infection (Bhattacharyya and Jha, 2012; Lanteigne *et al*., 2012). *Bacillus* spp. also produce various antibiotics, such as polymyxins, cyclins and colistin, to defend plants against pathogens (Liu *et al*., 2018; Maksimov *et al*., 2011). BMs can produce lytic enzymes, including proteases, chitinases and lipases, to destroy the cell walls of bacterial and fungal pathogens (Chen *et al*., 2020b; Joshi, 2012; Karthika *et al*., 2020; Lanteigne *et al*., 2012).

QS is a process by which bacteria release and sense small molecules to regulate bacterial group behaviour (Moreno‐Gámez *et al*., 2023). BMs can secrete lytic enzymes to degrade N‐acyl‐homoserine lactone, a QS signalling factor, to strengthen plant defense (Awan *et al*., 2011). This strategy decreases pathogen toxicity and infectiveness; however, it cannot inhibit or kill the pathogens reported to consistently exist in Gram‐negative bacteria, such as *Agrobacterium tumefaciens*, *Ralstonia solanacearum*, *Pseudomonas nigrifaciens* and *Ochrobactrum*. Iron is an essential element for both plants and microbes. BMs compete with pathogens for iron by biosynthesizing abundant high‐affinity siderophores to inhibit the growth of siderophore‐dependent pathogens and prevent pathogen infections (Saha *et al*., 2016; Shao *et al*., 2023; Sivasakthi *et al*., 2014; Verbon *et al*., 2017). For example, *B. subtilis* CAS15 can secrete the siderophore catecholate to inhibit the growth of several pathogens, including *Fusarium* spp., *B. anthracis*, *Pythium* spp., *Trichoderma* spp. and *Phytophthora* spp. (Yu *et al*., 2011).

BMs can induce plants to secrete bacteriostatic compounds. For example, *B. subtilis* enhances tobacco resistance against bacterial pathogens by improving plant flavonoid accumulation (Nazari *et al*., 2017; Wang *et al*., 2022). BMs can also utilize plant immune systems to restrict infection (Nobori *et al*., 2020). Induced systemic resistance (ISR) is a crucial mechanism for enhancing immunity through JA and ET in plants (Yu *et al*., 2022). Four BMs, namely *Stenotrophomonas* sp., *Rhizobium* sp., *Ochrobactrum* sp. and *Advenella* sp., can activate ISR in *Astragalus mongholicus* once the plant is infected by *F. oxysporum* (Li *et al*., 2021). When the roots of carnation were inoculated with *Pseudomonas* sp. strain WCS417r prior to *Fusarium oxysporum f*. sp. *dianthi*, the number of diseased plants in all experiments with cultivar Pallas was reduced from about 50 to 20% (Van peer et al., 1991). *Pseudomonas putida*, a maize BM, can trigger ISR in the crop to defend against *Colletotrichum graminicola* (Neal and Ton, 2013; Planchamp *et al*., 2015).

### BMs help plants counter abiotic stresses

In response to abiotic stresses, plants secrete chemical signals and nutritional sources to recruit BMs and modify their microbiota. These BMs contribute to plant health through various mechanisms, such as producing phytohormones, solubilizing nutrients and modulating stress‐related gene expression.

Drought is detrimental to agriculture, and soil salinization poses a significant risk to agricultural output worldwide. The high osmotic environment causes the plant to absorb water and is blocked, resulting in a state of ‘physiological drought’, which seriously restricts the growth and yield of crops (Tarolli *et al*., 2024; Zhu, 2002). BMs enhance drought or salinity stress tolerance by producing various phytohormones, such as IAA, GA, CTK and ABA (Figure 2b). For example, *Azospirillum brasilense* produces IAA, which increases root length and biomass to improve water uptake in drought (Dodd *et al*., 2010). BMs can also produce ACC deaminase to reduce ET levels in plants (Carvajal *et al*., 2024; Tiwari *et al*., 2018). Certain BMs produce butanediol, which can induce stomatal closure, reduce water loss and improve drought tolerance (Cho *et al*., 2008). BMs produce EPSs, which improves soil aggregation and moisture retention (Bashan *et al*., 2004). EPSs also promote the formation of biofilms around plant roots. These biofilms can trap Na^+^, preventing them from entering the plant roots (Li *et al*., 2024). BMs can produce osmolytes, such as proline, which stabilize proteins and membranes and protect plants against drought and osmotic stress (Iqbal *et al*., 2014). Inoculation with *Azospirillum* and *Pseudomonas* has been used to increase proline levels in wheat and maize to improve drought tolerance (Nawaz *et al*., 2020). BMs stimulate the production of antioxidant enzymes, including superoxide dismutases, catalase and peroxidases. These enzymes scavenge reactive oxygen species (ROS) generated under stress conditions, reducing oxidative damage and enhancing plant resilience to drought and salinity (Lastochkina *et al*., 2017; Yogendra, 2015). Gene expression studies have shown that BMs inoculation can regulate the expression of vital stress‐responsive genes in plants, such as those encoding antioxidant enzymes, heat shock proteins and osmoprotectants, to enhance their ability to withstand drought (Ali *et al*., 2022; Mishra *et al*., 2020).

BMs can also help plants counter other abiotic stresses, including suboptimal light, circadian clock, CO_2_, temperature, humidity, water and nutrient conditions. By fostering a healthy rhizosphere microbiome (Zhu *et al*., 2023), BMs can also enhance plant resilience to these stresses, improve plant health and boost agricultural productivity.

## BMs regulate growth‐defense trade‐offs under biotic stresses via receptor pathways

As previously outlined, BMs promote growth by improving nutrient uptake, producing growth‐promoting hormones and stimulating root development; they also enhance disease resistance and help plants counter abiotic stresses. The subsequent section describes advancements in the current understanding of the involved receptor pathways.

### BMs regulate growth‐defense trade‐offs through the leucine‐rich repeat receptor‐like kinase (LRR‐RLK) pathway

**Figure 3:**
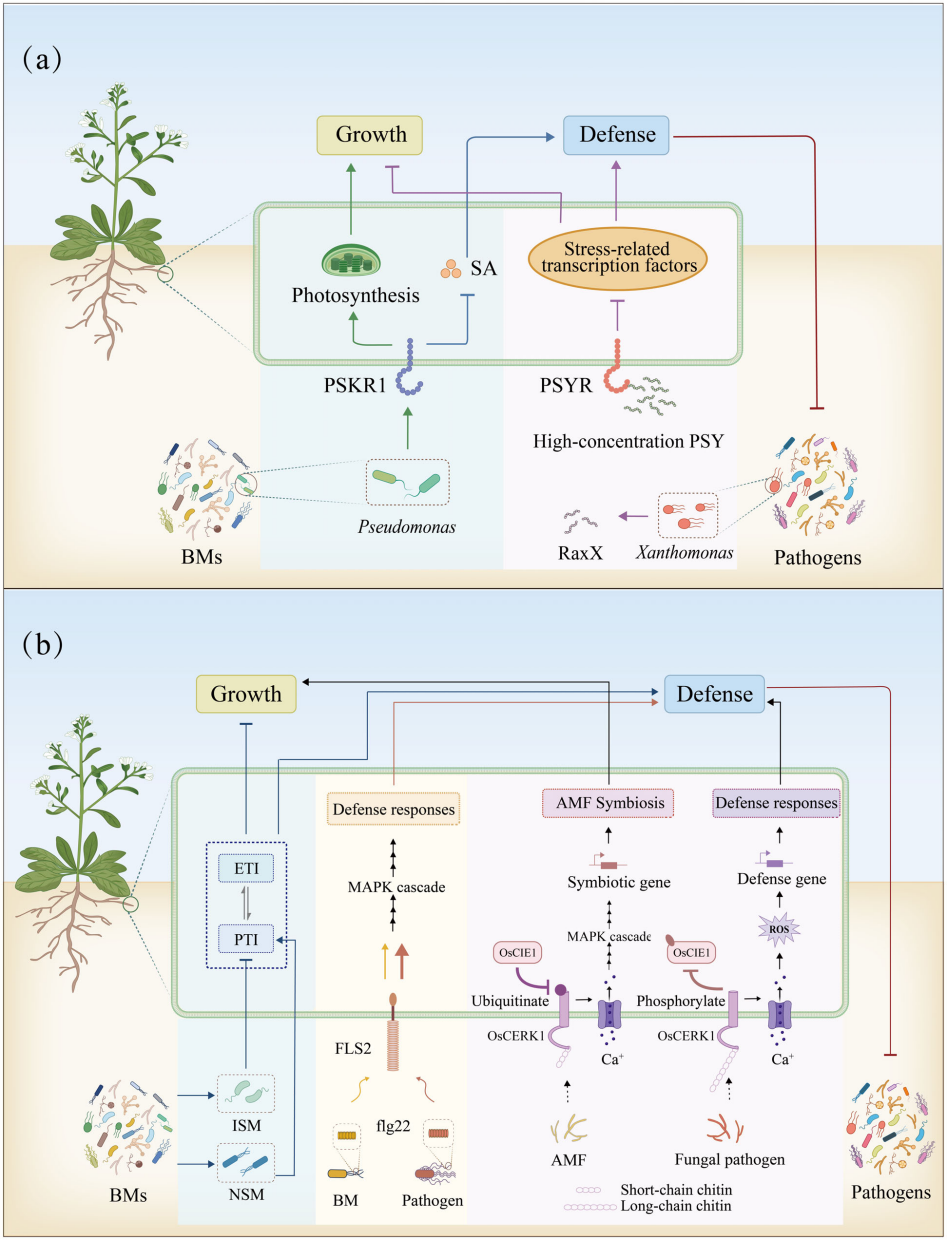
BMs are involved in growth‐defense trade‐offs in plants under biotic stresses. (a) BMs regulate growth‐immunity trade‐offs in plants through the leucine‐rich repeat receptor‐like kinase (LRR‐RLK) pathway; (b) BMs coordinate growth‐defense trade‐offs in plants through microbe‐associated molecular patterns (MAMPs). SA, Salicylic acid; PSKR1, Phytosulfokine receptor 1; PSY, Plant peptide containing sulphated tyrosine; PSYR, PSY's receptor; RaxX, a PSY1‐like sulphated peptide; PTI, Pattern‐triggered immunity; ETI, Effector‐triggered immunity; ISM, Immunity suppressive microbiome; NSM, Non‐suppressive microbiome; MAPK, Mitogen‐activated protein kinase; ROS, Reactive oxygen species; AMF, Arbuscular mycorrhizal fungi.

LRR‐RLK is the largest group of receptor‐like kinases in plants and includes various members that are crucial in growth, development and defense responses. Among the family, the phytosulfokine receptor 1 (PSKR1) is a receptor for phytosulfokines, which are a family of sulphated plant peptide hormones. Phytosulfokines are important in promoting cellular proliferation and enhancing plant growth. PSKR1 expression in plant roots is specifically induced by the presence of *Pseudomonas fluorescens* (Figure 3a). This indicates a potential evolutionary adaptation by plants to optimize interactions with these common soil bacteria, which are integral components of the rhizosphere microbiome. The presence of *Pseudomonas* triggers PSKR1 expression and induces significant physiological responses that balance plant growth and defense by promoting photosynthesis and root development. PSKR1 expression also suppresses defense mechanisms by inhibiting the SA‐dependent immunity response (Song *et al*., 2023).

A study by Ogawa‐Ohnishi et al. revealed that plant peptide containing sulphated tyrosine (PSY) and its receptor (PSYR) are crucial for mediating the trade‐off between growth and stress response in *Arabidopsis* (Ogawa‐Ohnishi *et al*., 2022). PSYR also belongs to the LRR‐RLK family. *Xanthomonas oryzae* produces a PSY1‐like sulphated peptide, RaxX (Pruitt *et al*., 2017). The PSY‐PSYR signalling axis manages the trade‐off between plant growth and stress responses by modulating transcription factor activity as follows. When PSY peptide levels are decreased, the receptors activate stress‐related transcription factors, favouring stress responses rather than growth. Conversely, in the presence of sufficient PSY peptides, these transcription factors are suppressed, reducing stress responses and favouring growth and development (Ogawa‐Ohnishi *et al*., 2022). These observations underscore the critical role of plant‐sulphated peptide receptors in the universal framework that regulates plant reactions to various stressors; however, the specific molecules secreted by *Pseudomonas* to regulate PSKR1 remain unknown.

### BMs coordinate growth‐defense trade‐offs through microbe‐associated molecular patterns (MAMPs)

MAMPs are conserved molecular structures found across various microbes, including bacteria, fungi and viruses. PRRs on the surfaces of plant cells facilitate the recognition of MAMPs. These receptors bind MAMPs, which initiates MAMPs‐triggered immunity (MTI). MTI activation exhibits a negative effect on plant growth (Huot *et al*., 2014; Zipfel *et al*., 2004). Many hyper‐immune mutants are stunted, suggesting that inducing immune responses is energetically expensive for plants (Huot *et al*., 2014; Zipfel *et al*., 2004).

The rhizosphere microbiome assists plants in regulating MTI. Within the rhizosphere, microbiomes can be categorized into two types, namely the immune‐suppressive and non‐suppressive types (Figure 3b). When the proportion of immunity suppressive microbiomes (ISMs) exceeds that of non‐suppressive microbiomes (NSMs), the rhizosphere microbiome primarily functions to increase growth; otherwise, it promotes immune responses (Ma *et al*., 2021). ISMs inhibit plant immunity by downregulating genes involved in the immune response, modulating host susceptibility to pathogens. This strategy prevents plants from mounting an excessive immune response to pathogens, which can impair growth. This conceptual framework is termed the ‘rheostat model’ (Ma *et al*., 2021).

flg22, a 22‐amino acid peptide found in bacterial flagellin, is a well‐studied MAMP that modulates MTI through sophisticated signalling networks (Liu, Huang *et al*., 2022). Flagellin Sensing 2 (FLS2) receptors on the plant cell membrane specifically recognize and bind to the flg22 fragment (Wang et al., 2023). This interaction leads to formation of the FLS2/BAK1 complex, which activates the MAPK cascade, causing ET production and defense‐related gene expression. flg22 proteins of specific BMs can also be recognized by plants; however, they stimulate weaker immune responses. For example, the *Burkholderia phytofirmans* strain PsJN is a known endophytic BM of potato, tomato and grapevine plants (Mitter *et al*., 2013). The recognition of *B. phytofirmans* through flg22 in grapevines triggered only a minor oxidative burst, weak and transient defense gene induction, and no growth inhibition. *B. phytofirmans* flg22 also elicited weaker immune responses than pathogenic *Pseudomonas aeruginosa* and *Xanthomonas campestris* (Trdá *et al*., 2014). This selective attenuation of immunity represents a refined strategy of growth‐defense trade‐off.

OsCERK1 is a crucial receptor kinase involved in MAMP cascades in plants. OsCERK1 recognizes and binds both short‐chain chitin from AMF and long‐chain chitin from pathogens (Petutschnig *et al*., 2014). Upon binding to short‐chain chitin, OsCERK1 triggers calcium influx and MAPK signalling to promote symbiotic gene expression and create favourable conditions for AMF infection and colonization (Miyata *et al*., 2014). Conversely, a complex between OsCERK1 and long‐chain chitin from pathogens can activate a stronger immune response. This response triggers ROS generation and defense gene expression, thereby protecting plants against pathogens (Wang *et al*., 2024). OsCERK1 is regulated by OsCIE1 (a U‐box E3 ligase) to prevent excessive autoimmunity (Miyata *et al*., 2014). Upon pathogen invasion, OsCERK1 phosphorylates OsCIE1 to block its activity and generate a strong immune response. Once pathogens are cleared, OsCIE1 re‐ubiquitinates OsCERK1 to reduce its activity and restore immune balance. These findings suggest that AMF and fungal pathogens can regulate the balance between plant growth and defense through the OsCERK1‐OsCIE1 pathway.

## BMs regulate growth‐defense trade‐offs under abiotic stresses

Besides balancing growth‐defense trade‐offs under biotic stresses, plants also face various abiotic stresses. BMs play a critical role in modulating plant hormone levels and activating stress response pathways.

### BMs coordinate growth‐defense through ABA Signalling

**Figure 4:**
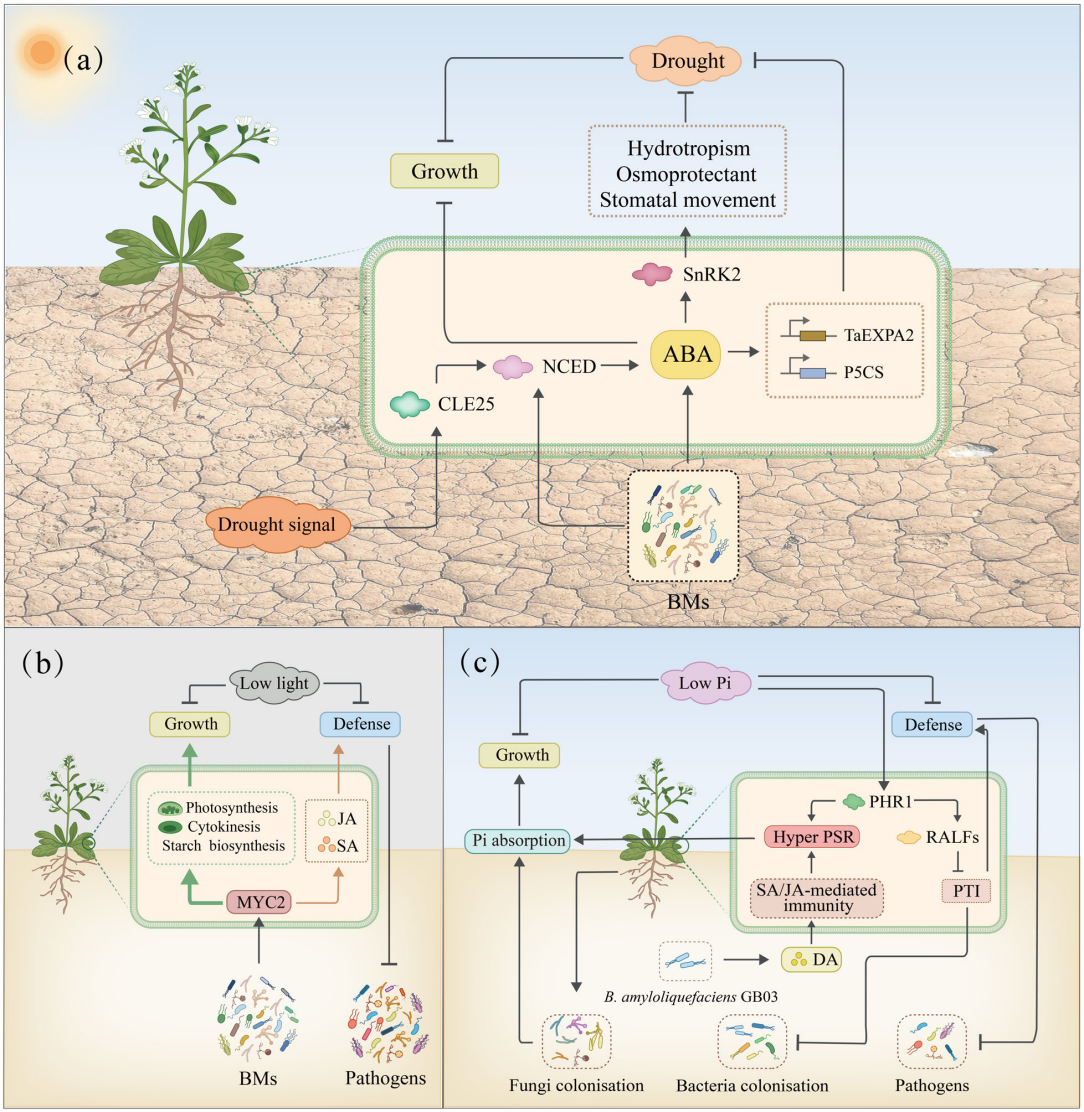
BMs are involved in growth‐defense trade‐offs in plants under abiotic stresses. (a) Under drought stress, BMs drive growth‐defense trade‐offs in plants through the ABA‐dependent pathway; (b) Under low light, BMs regulate growth processes (such as photosynthesis, cytokinesis, and the biosynthesis of starch and other sugars) and immune defenses (JA/SA‐mediated immunity response) in plants through the transcriptional regulator MYC2; (c) Under low P, beneficial rhizosphere fungi are recruited to help plants absorb P nutrients. BMs produce DA to induce PSR, which drives the growth‐defense trade‐off in plants. ABA, Abscisic acid; NCED, 9‐cis‐epoxycarotenoid dioxygenase; SnRK2, Sucrose non‐fermenting 1‐Related Protein Kinase 2; *TaEXPA2*, an α‐expansin gene; *P5CS*, gene encoding the key enzyme responsible for proline synthesis in plants; JA, Jasmonic acid; SA, Salicylic acid; DA, Diacetyl; PSR, P starvation response; PHR1, transcriptional regulator; RALFs, Rapid alkalinisation factors; PTI, Pattern‐triggered immunity.

ABA is central to regulating plant growth and development (Finkelstein, 2013). It also modulates defense against abiotic stresses by activating the phosphorylation of sucrose non‐fermenting 1‐Related Protein Kinase 2 (SnRK2) under drought conditions. SnRK2 phosphorylation promotes hydrotropism, osmoprotectant secretion and stomatal closure (Okamoto *et al*., 2013) (Figure 4a). The involvement of ABA in growth regulation and defense activation exemplifies the classic growth‐defense trade‐off, where resource allocation to one function can detract from another. When ABA induces stomatal closure to conserve water during drought, this response can also limit the ability of the plant to absorb carbon dioxide, reducing photosynthetic activity and potentially limiting growth (Verslues *et al*., 2006).

In plants, ABA is synthesized by the ABA synthase NCED. AMF and *Piriformospora indica* improve plant drought tolerance by inducing NCED expression. *A. brasilense*, a bacterium that has been used as a biofertilizer to promote plant growth, can produce ABA *in vitro* in a chemically defined medium (Bashan and de‐Bashan, 2010). Other bacteria have been reported to regulate ABA levels in plants. For example, *Streptomyces pactum* increases the ABA content of wheat leaves in order to upregulate the expression of drought resistance‐related genes, such as *TaEXPA2* and *P5CS* (Chen *et al*., 2016; Su *et al*., 2011). A transcriptomic analysis showed that *Piriformospora indica* upregulates the expression of ABA and related genes and improves host drought tolerance after it colonizes the corn rhizosphere (Li *et al*., 2023; Zhang *et al*., 2018). *Pseudomonas argentinensis* SA190, a root endophytic desert bacterium, enhances drought stress tolerance in *Arabidopsis* by priming the promoters of target genes in an epigenetic ABA‐dependent manner (Alwutayd *et al*., 2023). *B. cereus* NJ01 triggers SA‐ and ABA‐mediated immune responses against bacterial pathogens via the EDS1‐WRKY18 pathway (Wang, Wei *et al*., 2024). BMs can participate in growth‐defense trade‐offs in plants via ABA‐dependent pathways under stress; however, the underlying molecular mechanisms require further investigation.

### BMs integrate growth‐defense trade‐offs by regulating transcriptional factors

MYC2 is a pivotal transcriptional factor primarily functioning within the JA signalling pathway, which is crucial for plant defense responses. MYC2 also regulates the cross‐talk between JA and other plant hormones (SA, ABA, ET, AUX, CTK, GA and BRs) and mediates the growth‐defense trade‐off by regulating photosynthesis, cytokinesis, and the biosynthesis of starch and other sugars to promote plant growth (Figure 4b). *P. fluorescens* WCS417r, a beneficial root microbe, can activate MYC2 (Kazan and Manners, 2013). MYC2 can suppress growth‐related genes to allocate resources toward defense under certain conditions. Conversely, under low‐light stress conditions, MYC2 activity can be transformed by BMs to support growth. This mechanism is well‐supported by reports discussing the role of MYC2 in priming enhanced defense responses during rhizobacterium‐induced systemic resistance in *Arabidopsis* (Pieterse *et al*., 2014; Pozo *et al*., 2008). MYC2 interacts with pathways influenced by low‐light stress, effectively linking environmental conditions to plant growth and defense strategies. Under normal‐light conditions, these root microorganisms enhance both growth and immune defense. In contrast, under low‐light conditions, they primarily promote growth. This regulatory effect in *Arabidopsis* is also mediated by MYC2, which orchestrates plant responses to varying environmental stimuli by modulating gene expression (Hou *et al*., 2021); however, similar to the microbe‐LRR‐RLK axis, the molecular mechanism by which microbes regulate MYC2 remains unknown.

Phosphate starvation response 1 (PHR1) is a master transcriptional factor in P signalling and is responsible for activating genes involved in uptake and homeostasis under P‐deficient conditions. PHR1 is crucial in growth‐defense trade‐offs based on phosphate availability. Under phosphate‐sufficient conditions, PHR1 suppression by OsSPX1/2 causes reduced phosphate starvation responses, allowing the plant to allocate more resources toward growth (He *et al*., 2024). Conversely, under phosphate‐deficient conditions, PHR1 activation favours the enhancement of defense mechanisms (Puga *et al*., 2014) (Figure 4c). BMs can cooperatively regulate PHR1‐related genes. *B. amyloliquefaciens* GB03 can produce diacetyl (DA) (Ma *et al*., 2022), a microbial signal that influences the plant's phosphate starvation response, with PHR1 being a crucial mediator. Under P starvation, *B. amyloliquefaciens* enhances DA production to enhance P uptake in plants with hyper‐PSR; however, under P‐sufficient conditions, DA inhibits the production of plant ROS and enhances self‐colonization without affecting plant disease resistance (Morcillo *et al*., 2020; Zhang, 2023). Under P deficiency, beneficial rhizosphere fungi are recruited and colonize the roots to improve P absorption, while bacterial colonization is blocked to avoid P competition. Under P‐sufficient conditions, beneficial rhizosphere bacteria are recruited, while fungal invasion is prevented by Trp‐derived metabolites (Hiruma *et al*., 2016; Morcillo *et al*., 2020). These findings highlight a sophisticated regulatory network which is crucial for optimizing fitness across varying P conditions.

Specific small peptides are involved in plant growth and development, stress response and plant‐microbe interactions (Chen *et al*., 2020a; Katsir *et al*., 2011; Stührwohldt and Schaller, 2019). For example, PSY, CLE25 and CLE14 are involved in biotic (Ogawa‐Ohnishi et al., 2022), drought (Takahashi *et al*., 2018) and low P stresses (Gutiérrez‐Alanís *et al*., 2017), respectively. Many pathogenic bacteria can produce small peptide analogues, which are recognized by plants as real signals. These peptide analogues activate downstream growth, development and immune defense pathways to protect against the invasion of pathogenic bacteria (Zhang *et al*., 2020). These peptides can also be developed as auxiliaries to help BMs participate in the regulation of growth and development in plants.

Rhizosphere microbiomes assist plants in defending against pathogenic bacteria and contribute to the regulation of host immune responses through various mechanisms. Rhizosphere microbiomes help plants balance energy allocation between immune responses and adaptability to minimize growth loss under pathogenic infection. In addition to the abovementioned molecules, VOCs, small peptides, LPS and siderophores secreted by microbes can enhance plant biomass and root growth while also increasing tolerance to copper stress and bacterial pathogens (Chiang *et al*., 2024; Huot *et al*., 2014; Ku *et al*., 2024); however, the detailed components of these molecules and the mechanisms by which they regulate growth‐defense trade‐off in plants require further elucidation.

## Concluding remarks and future perspectives

The rhizosphere microbiome has been termed the ‘second plant genome’ and can functionally affect plant fitness (Xun *et al*., 2021). This review summarizes current studies on the role of BMs in plant growth, biotic/abiotic stresses and growth‐stress trade‐offs. Although substantial evidence has been provided, many of the underlying mechanisms remain unclear. Most of the existing research utilizes individual strains. However, how SynComs or more complex rhizosphere microbiomes coordinate under natural conditions is unclear. The signal perception receptors and pathways for growth‐defense trade‐offs in plants are partially elucidated and warrant further research.

**Figure 5:**
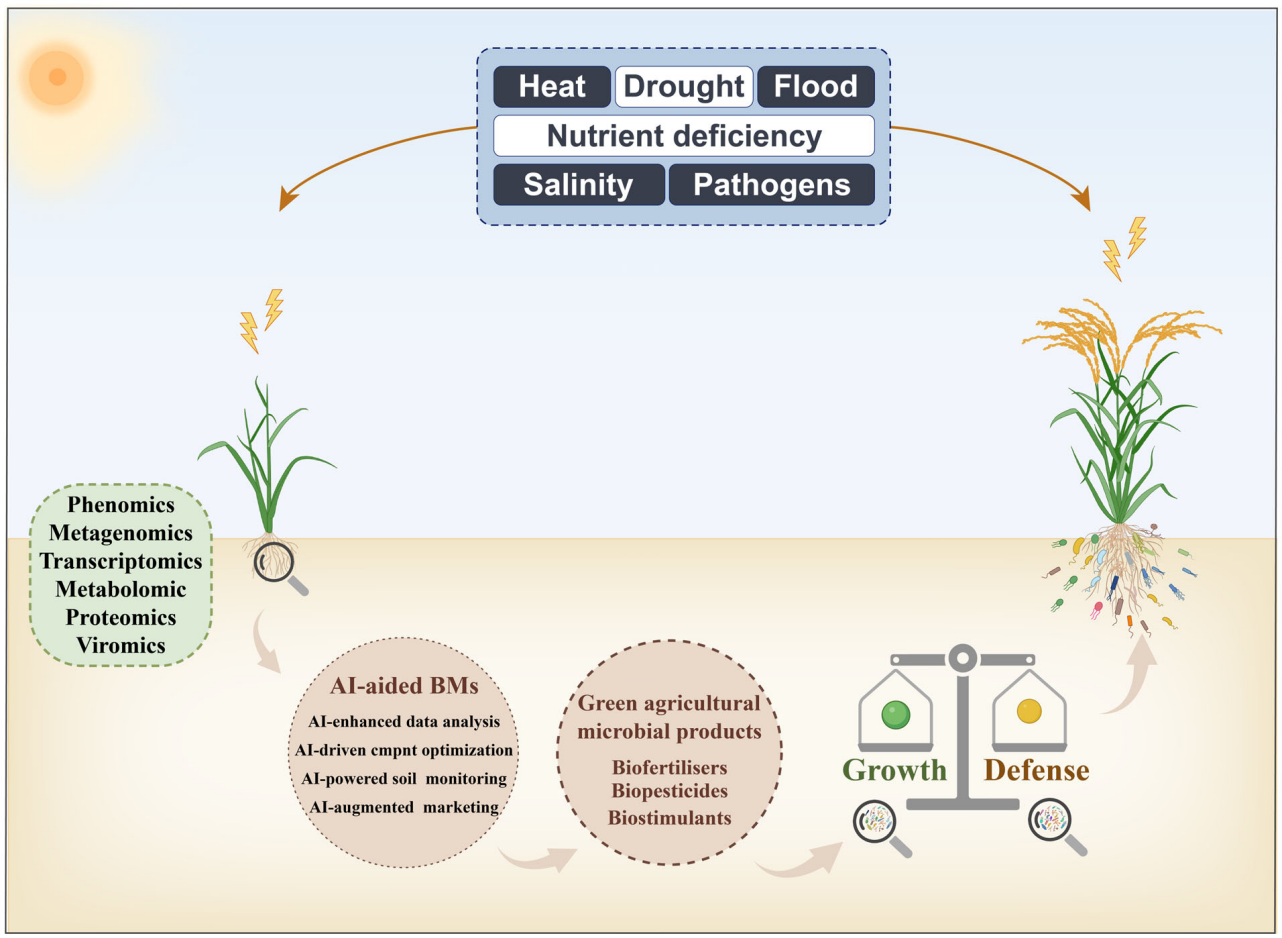
AI‐aided BMs research strategies and further applications in agriculture. AI can help elucidate the interaction patterns among plant genotypes, phenotypes, and BMs by enhancing data analysis, component optimization, soil monitoring, and marketing. This approach promotes the development and application of biofertilizers, biopesticides, and biostimulants. By balancing growth‐defense trade‐offs, AI can help improve crop yield and disease resistance. AI, Artificial intelligence.

The integration of artificial intelligence (AI) into microbiome research and biofertilizer development has significantly accelerated research advances and commercialization efforts (Figure 5). AI algorithms are adept at handling large and complex datasets, making them ideal for microbiome research (Pu *et al*., 2024). They can analyse omics data (metagenomics, transcriptomics, proteomics and viromics) to identify microbial communities and understand their interactions with plant hosts. Compared with bioinformatics tools, generative AI models can be used to create predictive models that forecast how microbial communities will behave under different environmental conditions (Hernández Medina *et al*., 2022). This is particularly useful for understanding how biofertilizers will perform in complex and diverse agricultural settings. AI‐driven optimization algorithms can select the most beneficial microbial strains to fine‐tune the composition of biofertilizers. These algorithms can also integrate variables such as nutrient availability, soil type and crop requirements to develop tailored biofertilizer solutions (Osman *et al*., 2023). AI‐powered sensors and IoT devices also make the real‐time monitoring of soil health and plant growth, which provides continuous feedback and allows for the dynamic adjustment of biofertilizer applications based on field conditions (Qazi *et al*., 2022). AI models can also offer insights that aid in the commercialization of biofertilizers by analysing market trends, customer preferences and competitive landscapes (Makepa and Chihobo, 2024). In summary, AI can accelerate microbiome research and biofertilizer development by handling complex data, optimizing formulations, enabling real‐time monitoring and aiding in market analysis.

Agriculture is critically threatened by intensifying climate change, natural disasters and decreased biodiversity (including microbial diversity). Since BMs are involved in plant adaptation to the environment, exploring the natural plant microbiome is a promising approach to increase crop yield in adverse environments (Mazza Rodrigues and Melotto, 2023). Green biological products, such as biofertilizers, biopesticides and biostimulants, are more environmentally friendly and effective than chemical fertilizers and pesticides. The global microbial fertilizer market size exceeded US$2.9 billion in 2020 and is expected to reach US$5.2 billion in 2031. The demand for microbial fertilizers is currently the highest in North America, followed by Europe, Asia Pacific and Latin America. Owing to incentives, such as tax exemptions and subsidized inputs, the production and consumption of microbial fertilizers are growing in countries such as Canada, Argentina, China, India, Europe and the United States (http://www.marketsandmarkets.com/Market-Reports/compound-biofertilizers-customized-fertilizers-market-856.html). The functions of existing microbial fertilizer products include but are not limited to improving nutrient absorption and utilization efficiency, stimulating plant root growth, preventing and controlling plant diseases and pests and improving plant stress resistance. BMs are essential resources for developing biofertilizers. However, obtaining BMs and optimizing them for commercial use has been challenging. According to current studies, BMs used as biofertilizers have several problems, including variable effectiveness, short shelf life, slow action, quality control issues and compatibility with certain chemical fertilizers or pesticides, et al. (Bharti and Suryavanshi, 2021; Mahanty *et al*., 2017; Malusà *et al*., 2016; Shravani, 2019). This topic has received significant attention in the agricultural field (Gfeller *et al*., 2023). Human and animal microbiome studies, multiomics, large models and bioengineering efforts (Bai *et al*., 2023; Cammarota *et al*., 2020; Lin *et al*., 2023; Parizadeh and Arrieta, 2023) have aided in further advancing the use of BMs in large‐scale agriculture. A new concept, ‘rhizosphere synbiotic’, consisting of core functional rhizosphere microorganisms and their beneficial substances (postbiotics) has also been developed. The related study has provided a theoretical guide for developing novel regulatory strategies to reduce soil pathogens (Yang *et al*., 2023); however, the environment of plant microbiomes is more open and complex, warranting further investigation.

## Author contributions

YL and AS created the figures. YL, BJ and AS wrote the draft of the manuscript; YC, ZX, YL, YY, YW and BJ revised the manuscript. YL, YC and BJ performed the revision of the manuscript.

## Declaration of interests

The authors declare no competing interests.

## Data Availability

Data sharing not applicable to this article as no datasets were generated or analysed during the current study.
